# Two cases of type 2 diabetes mellitus in elderly patients successfully treated with monthly exenatide long‐acting release

**DOI:** 10.1002/ccr3.2139

**Published:** 2019-04-07

**Authors:** Kenchi Takenaka, Rie Nishitani

**Affiliations:** ^1^ Internal Medicine Jinai Clinic Kawaguchi‐Shi Japan

**Keywords:** elderly, exenatide long‐acting release, monthly, type 2 diabetes mellitus

## Abstract

Two elderly patients with poorly controlled type 2 diabetes mellitus had difficulty self‐managing their medications. Exenatide long‐acting release (LAR), with an extended administration interval of 1 month, maintained hemoglobin A1c (HbA1c) level in the 7% range. Monthly administration of exenatide‐LAR may be considered for use in carefully selected elderly individuals.

## 1 INTRODUCTION

The number of elderly patients with type 2 diabetes mellitus has increased.^1^ Elderly patients have an increased risk of drug‐related adverse events and hypoglycemia.^1^ They usually take multiple medications, with their ability to manage medications often compromised by reduced cognitive function. Therefore, drug therapy should have a low risk of hypoglycemia and be as safe and simple as possible.

Exenatide, a glucagon‐like peptide 1 receptor agonist (GLP‐1RA), has high efficacy in patients with type 2 diabetes mellitus,^2^ with low risk of hypoglycemia when used as a single agent. A long‐acting release (LAR) formulation of exenatide (exenatide‐LAR) is effective when administered once weekly, making it attractive to patients and caregivers. Moreover, extending the administration interval may be possible once blood glucose levels have stabilized. We report this administration in elderly patients with poorly controlled type 2 diabetes mellitus who had difficulty self‐managing medications.

## 2 CASE REPORTS

### 2.1 Case 1

A 90‐year‐old woman was diagnosed with type 2 diabetes mellitus more than 10 years ago. Her body weight was 41.7 kg (BMI: 20.1 kg/m^2^). There was no problem with liver function, AST: 24 (10‐40) IU/mL, and renal function, creatinine: 0.68 (0.45‐0.82) mg/dL. Diabetic complications were not observed. Initially, hemoglobin A1c (HbA1c) level was controlled at 6% by glimepiride (3 mg) and metformin (250 mg); however, she visited our clinic before the scheduled date claiming that “the medicine is gone.” Due to cognitive decline, an overdose of glimepiride and metformin was suspected, and the drugs were discontinued.

**Figure 1:**
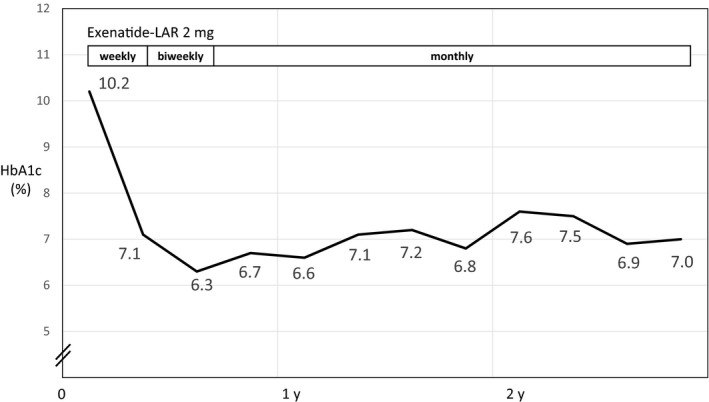
A 90‐year‐old woman had type 2 diabetes mellitus (Case 1). After beginning once weekly exenatide‐LAR (2 mg) treatments, the hemoglobin A1c (HbA1c) level rapidly declined. After extending the interval between treatments to a month, the HbA1c level was maintained in the 7% range for more than 2 y

The patient was prescribed teneligliptin (20 mg), a dipeptidyl peptidase 4 (DPP‐4) inhibitor with low hypoglycemia risk, but HbA1c levels increased to 10.2% within 6 months. Although resuming glimepiride treatment was considered, we opted to administer 2 mg exenatide‐LAR once weekly to minimize the risk of hypoglycemia caused by overdosing. Despite declining cognitive function, she visited our clinic weekly for exenatide‐LAR injections. HbA1c level rapidly decreased; after 4 months, it reached 7.1% (Figure 1). Moreover, good blood glucose control was achieved.

To reduce the dose frequency, we extended the dose interval to 2 weeks and subsequently to 1 month. HbA1c level was in the 6% range when exenatide‐LAR was administered every 2 weeks. It was in the 7% range for more than 2 years when administered once monthly. Fasting glucose just before the next injection did not rise. Once‐a‐month administration allowed the patient to easily receive effective outpatient treatment despite cognitive loss. However, outpatient treatment became difficult after the patient sustained a spinal compression fracture; hence, the drug was administered by a home care provider without issues. During the period of observation, liver and renal functions were not changed. The patient's body weight did not appreciably change, and no adverse events, such as loss of appetite or hypoglycemia, were observed with exenatide‐LAR.

### 2.2 Case 2

An 85‐year‐old woman was diagnosed with type 2 diabetes mellitus several years ago. Her body weight was 61.0 kg (BMI: 31.1 kg/m^2^). There was no problem with liver function, AST: 24 (10‐40) IU/mL, and renal function, creatinine: 0.54 (0.45‐0.82) mg/dL. Diabetic complications were not observed. She was treated with teneligliptin (20 mg), but blood glucose control gradually worsened and HbA1c level increased to 9.7%. Her family informed us that she often forgot to take her multiple medications, which we assumed accounted for the poor blood glucose control.

**Figure 2:**
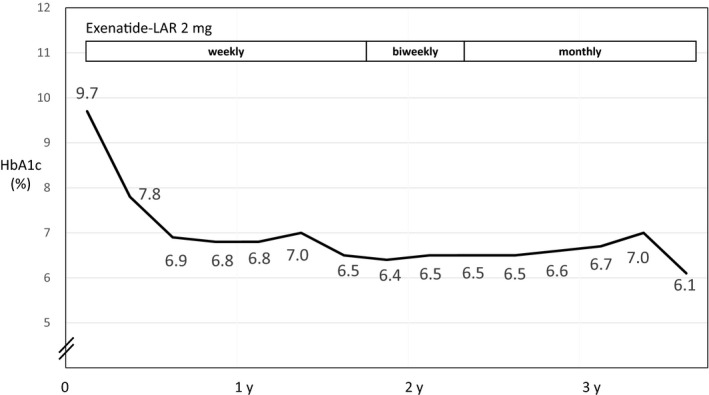
An 85‐year‐old woman had type 2 diabetes mellitus (Case 2). After beginning once weekly exenatide‐LAR (2 mg) treatments, the hemoglobin A1c (HbA1c) level rapidly declined. After extending the interval between treatments to a month, the HbA1c level was maintained in the 7% range for more than 1 y

As the patient did not wish to receive at‐home injections, weekly injections of 2 mg exenatide‐LAR were administered at our clinic. After switching from teneligliptin to exenatide‐LAR, HbA1c level rapidly decreased and was maintained in the 6% range (Figure 2). The administration interval was extended to every 2 weeks and subsequently to 1 month. The blood glucose level was well controlled after both extensions. It remained steady for more than 1 year after shifting to monthly administration even though the patient underwent chemotherapy for breast cancer during this time. Only exenatide‐LAR was required for blood glucose control. During the period of observation, liver and renal functions were not changed. The patient's body weight did not appreciably change, and no adverse events, such as loss of appetite or hypoglycemia, were observed.

## 3 DISCUSSION

We presented two cases of type 2 diabetes mellitus in elderly patients who could not self‐manage medications. Weekly administration of exenatide‐LAR reduced blood glucose levels, which were maintained by monthly administration. This is the first report of successful monthly exenatide‐LAR treatment.

Exenatide‐LAR is encapsulated in microspheres made of biodegradable polymers, which enables gradual, long‐term release via diffusion and subsequent erosion of the polymers. Blood concentration of exenatide‐LAR peaks 7 weeks and remains high for 12 weeks by single administration.^3^ The long retention time enables monthly administration in patients with well‐controlled blood glucose levels.

Since DPP‐4 inhibitors are highly effective, especially in East Asians,^4^ and very safe, their use has recently increased in Japan.^5^ Owing to their safety, they are widely used in elderly patients with type 2 diabetes mellitus. However, when DPP‐4 inhibitors are insufficient, GLP‐1RAs are administered to increase effectiveness.^6^ Similar to DPP‐4 inhibitors, GLP‐1RAs have low hypoglycemia risk when used alone. Although they are a valid treatment option for elderly patients, side effects such as nausea and vomiting are possible. Moreover, because GLP‐1RAs are injected, the administration is more complicated than oral medications and might cause slight pain.

Elderly patients are often incapable of self‐managing their medications due to cognitive decline and require assistance from caregivers. Extending the interval between exenatide‐LAR injections from 1 week to 1 month after achieving blood glucose control is beneficial, as it reduces the number of clinic visits and medications required per month, improving self‐management, and lessens the workload of caregivers and clinic staff. Thus, even when self‐management is challenging, monthly exenatide‐LAR provides reliable medical treatment, with minimal intervention by outsiders. Monthly exenatide‐LAR is cheaper than DPP‐4 inhibitors; thus, monthly exenatide‐LAR treatment reduces medical costs.

We described the effectiveness, free of hypoglycemia, and relative convenience of weekly exenatide‐LAR in elderly patients with type 2 diabetes mellitus who had difficulty self‐managing medications. We showed that extending the administration interval to 1 month did not diminish drug effectiveness. This reduces the burden on patients, caregivers, and clinic staff, as well as medical costs. As few drugs are effective and safe in elderly patients, exenatide‐LAR is potentially useful for the treatment of elderly patients with type 2 diabetes mellitus.

## CONFLICT OF INTEREST

None declared.

## AUTHOR CONTRIBUTION

KT: is the primary physician, treated the patients, and drafted the manuscript. RN: reviewed, revised, and supervised the drafting of the manuscript. All authors read and approved the final manuscript.

## References

[1] Kirkman MS , Briscoe VJ , Clark N , et al. Diabetes in older adults. Diabetes Care. 2012;35(12):2650‐2664. 23100048 10.2337/dc12-1801 PMC3507610

[2] Kadowaki T , Namba M , Imaoka T , et al. Improved glycemic control and reduced bodyweight with exenatide: a double‐blind, randomized, phase 3 study in Japanese patients with suboptimally controlled type 2 diabetes over 24 weeks. J Diabetes Investig. 2011;2(3):210‐217. 10.1111/j.2040-1124.2010.00084.x PMC4014921 24843486

[3] Fineman M , Flanagan S , Taylor K , et al. Pharmacokinetics and pharmacodynamics of exenatide extended‐release after single and multiple dosing. Clin Pharmacokinet. 2011;50(1):65‐74. 21142268 10.2165/11585880-000000000-00000

[4] Kim YG , Hahn S , Oh TJ , Park KS , Cho YM . Differences in the HbA1c‐lowering efficacy of glucagon‐like peptide‐1 analogues between Asians and non‐Asians: a systematic review and meta‐analysis. Diabetes Obes Metab. 2014;16(10):900‐909. 24655583 10.1111/dom.12293

[5] Seino Y , Kuwata H , Yabe D . Incretin‐based drugs for type 2 diabetes: focus on East Asian perspectives. J Diabetes Investig. 2016;7(Suppl 1):102‐109. 10.1111/jdi.12490 PMC4854513 27186364

[6] Brunton S . GLP‐1 receptor agonists vs. DPP‐4 inhibitors for type 2 diabetes: is one approach more successful or preferable than the other? Int J Clin Pract. 2014;68(5):557‐567. 24499291 10.1111/ijcp.12361 PMC4238422

